# Additive enhancement of wound healing in diabetic mice by low level light and topical CoQ10

**DOI:** 10.1038/srep20084

**Published:** 2016-02-02

**Authors:** Zhigang Mao, Jeffrey H. Wu, Tingting Dong, Mei X. Wu

**Affiliations:** 1 ShangHai 9th People Hospital, Shanghai Jiao Tong University School of Medicine, ShangHai, China; 2 Wellman Center for Photomedicine, Massachusetts General Hospital, Department of Dermatology, Harvard Medical School, Boston, MA 02114

## Abstract

Diabetes, a highly prevalent disease that affects 9.3% of Americans, often leads to severe complications and slow wound healing. Preclinical studies have suggested that low level light therapy (LLLT) can accelerate wound healing in diabetic subjects, but significant improvements must be made to overcome the absence of persuasive evidence for its clinical use. We demonstrate here that LLLT can be combined with topical Coenzyme Q10 (CoQ10) to heal wounds in diabetic mice significantly faster than LLLT alone, CoQ10 alone, or controls. LLLT followed by topical CoQ10 enhanced wound healing by 68~103% in diabetic mice in the first week and more than 24% in the second week compared with untreated controls. All wounds were fully healed in two weeks following the dual treatment, in contrast to only 50% wounds or a fewer being fully healed for single or sham treatment. The accelerated healing was corroborated by at least 50% higher hydroxyproline levels, and tripling cell proliferation rates in LLLT and CoQ10 treated wounds over controls. The beneficial effects on wound healing were probably attributed to additive enhancement of ATP production by LLLT and CoQ10 treatment. The combination of LLLT and topical CoQ10 is safe and convenient, and merits further clinical study.

There are an estimated 371 million cases of diabetes worldwide, putting patients at serious risk for wound healing complications that reduce both quality and length of life. In particular, foot ulcers hamper 15% of all diabetics, accounting for the majority of non-traumatic lower limb amputations in the United States^1^. These complex wounds result from a combination of diminished immune function, peripheral neuropathy, arterial occlusion, and miscellaneous factors such as limited joint mobility and improper footwear^1^,^2^,^3^. Small sores are readily healed in healthy individuals but frequently develop into deep ulcers, leading to infections of underlying tissues and bones that sometimes necessitate amputation in diabetic patients. Treatment of these chronic wounds in diabetic patients mainly relies on conventional wound bed preparation, mechanical and surgical debridement, and antibiotic therapy to battle infections^4^,^5^. However, these managements have extremely limited efficacy, leading to a high 5-year mortality rate following amputation^6^.

Considering the toll on patients and $11 billion cost to American health care payers^7^, there is a substantial demand for better approaches to diabetic wound management. The potential of low-level laser therapy (LLLT), a non-invasive treatment that some studies have shown to successfully accelerate wound healing in specific circumstances, is particularly attractive^8^. LLLT does not cause a noticeable temperature change in subjects, yet seems to stimulate tissue repair and relieve pain^8^,^9^,^10^. While their efficacy in clinical settings has long been disputed^11^, an increasing number of studies have contended that LLLT can increase fibroblast proliferation, collagen production, angiogenesis, granulation formation, and other positive effects^12^. As for diabetes specifically, a study proved that 633nm laser at 10 J/cm^2^ accelerated wound healing in diabetic rats by ~38%^1^. Systematic reviews have been hindered by lack of adequate sample sizes and consistent methodologies in clinical studies, but demonstrate plausibility and safety of using LLLT to treat diabetic wounds^2^.

Therapeutic mechanisms of LLL are not well understood to date. Considering that thermal increases are absent following LLLT, photobiomodulation may be the major contributor of the reported acceleration in tissue repair^8^,^12^,^13^. For example, one event suspected to contribute significantly to wound healing is the absorption of far red or near infrared light (600–1100 nm) by the redox active metal centers of cytochrome c oxidase, also known as complex IV of the electron transport chain at mitochondria^12^. Such absorption is thought to increase mitochondria respiration activity and ATP synthesis^8^,^13^. Likewise, ubiquinone CoQ10 can enhance mitochondrial activity by virtue of being a cofactor in the complexes I–III in the mitochondrial electron transport chain^14^,^15^. Besides, CoQ10 acts as a potent anti-oxidant and displays anti-ulcer potential^15^,^16^,^17^,^18^. Because LLL and CoQ10 target different complexes in the mitochondrial electron transport chain, these two modalities may additively accelerate diabetic wound healing.

With this in mind, we tested and found that LLLT followed by topical CoQ10 resulted in diabetic wound healing significantly faster than individual treatment or sham-treated controls. The dual treatment is simple, safe, and holds great promise for future management of diabetic wounds in the clinics.

## Materials and Methods

### Animals

The experimental subjects were 5-week-old male Swiss Webster mice (Charles river lab, USA) weighing 20–30 g. The mice were maintained in a temperature-controlled room under a 12 h light/12 h dark cycle and provided *ad libitum* access to food and water. All animal experiments were performed according to the National Institutes of Health guidelines for the Care and Use of Laboratory Animals and approved by the Subcommittee on Research Animal Care of the Massachusetts General Hospital.

### Animal model and study design

To induce diabetes in mice, streptozotocin (120 mg/kg, Sigma) in sodium citrate buffer (pH 4.0) was injected intraperitoneally after fast for 4~6 hr. Measurement with Blood Glucose Meter (Germaine Laboratories) confirmed diabetic state, which was conducted for three consecutive days after one week of streptozotocin injection and verified one day before surgical wounds. Mice exhibiting glucose levels over 200 mg/dl for all four tests were considered to be diabetic and enrolled to the study. On day 30 after streptozotocin injection, the animals were shaved on the dorsal skin after anesthetized by intraperitoneal administration of ketamine (90 mg/kg) /xylazine (10mg/kg). Two full-thickness, 1 cm^2 ^square defects were made on the thoracic and lumbar regions of each animal, respectively, one to receive a treatment and another to serve as a corresponding control such that the treatment and corresponding control could be compared on the same subject as listed in the Table 1. The 24 mice were randomly selected for different treatments with 12 wounds in each group. Half of the mice in each group were sacrificed for skin sampling on day 7 and the remaining mice were sacrificed on day 16. Control wounds experienced sham light and then topical vehicle (soybean oil) application, while the rest of the wounds received LLLT+ topical vehicle (LLLT), sham light +topical CoQ10 (CoQ10) or LLLT followed by topical CoQ10 (LLLT+CoQ10). LLLT was applied to the wound areas every two days starting in 24 hours after wound formation using an 830nm infrared light emitting diode (LED) from Omnilux new-U, USA. The average irradiance of the light was 14 mW/cm^2 ^for a total exposure time of 5 minutes or an energy density of 4.2 J/cm^2^ on the surface of the wound as measured by Handheld Laser Power & Energy Meter (Ophir Nova II). During the illumination, the mouse was covered by aluminum sheet with an opening area of 1 cm^2^ to expose the wound, at which LLL was directed vertically at a distance of 6 cm above. The sham light was administered similarly with a small soft white LED lightbulb (3W A15) from General Electric (GE) such that no heat was generated on the wound. The skin temperature during 5 minutes of illumination increased ≤0.1 °C with either light source. After illumination, CoQ10 was topically applied to the wound so that there was not any direct interaction between LLL and CoQ10. An over-the-counter CoQ10 supplement (30 mg/ml, Bio Quinone) was diluted by 5 times in soybean oil and 50 μl of the CoQ10 solution was gently wiped onto the surface of the wound immediately post-LLLT. The wound was covered 20 minutes later by a bandage to protect from mouse bites or scratching.

### Wound-healing measurement

Photographs of each full-thickness skin defect, next to a ruler for calibration, were taken using an Olympus digital camera. Wound area in square centimeters was analyzed using Image J software and repeated measurements were accurate within 2%. The first images were obtained on the day of injury (day 0). Subsequent images were captured on the third, seventh, and thirteenth days post-injury. The wound healing areas were measured by percentages of a healed area relative to an original wound area using the following formula: The healed area percentage = (original wound area – unepithelialized area)/original wound area^19^, where healing referred to a combination of wound constriction and epithelization, not including crust formation and granulation. The measurement was conducted in a manner blinded to the study groups.

### Cell proliferation assay

Cell proliferation *in situ* was analyzed with 5-ethynyl-2′-deoxyuridine (EdU) assays from Invitrogen. EdU is a thymidine analogue and incorporated into the DNA of dividing cells. Mice were injected intraperitoneally with 100 μg of EdU in PBS on day 6 after wounds and then the skin specimens were collected one day later^20^. Two specimens were excised from each wound, one from the center of the wound bed and the other from uninjured adjacent skin served as reference such that a total of 4 specimens were collected from each mouse. The skin specimens were formalin-fixed, embedded in paraffin, and cut into 5μm thick sections. For EdU staining, paraffin in the tissue sections was removed and the tissue was stained with the Click-iT^®^ EdU Alexa Fluor^®^ 488 Imaging Kit (Invitrogen, USA), counterstained with DNA dye DAPI (4′,6-diamidino-2-phenylindole) provided in the kit per the manufacturer’s instruction. The stained sections were imaged by confocal microscopy (Olympus FV1000, Olympus, Japan) and analyzed by Image J software. Percentages of EdU-positive cells relative to a total number of cells were obtained by manually counting the number of EdU-positive green cells and DAPI-positive blue cells in 100 microscopic fields randomly selected in each sample in a sample-blind manner.

### Hydroxyproline levels

Hydroxyproline levels were used to evaluate collagen content in the extracts of healing skin tissues. Specimens were excised and weighed from the wound bed and uninjured adjacent skin as above. The skin sample of 5 mg was homogenized in 100 μl of water before transfer to a pressure-tight vial with PTFE-lined cap and addition of 100 μl concentrated hydrochloric acid (HCl, ~12 M). It was then capped tightly and hydrolyzed at 120 °C for 3 hours. The resultant supernatant (20 μl) was transferred to a 96-well plate and placed in a 60 °C oven to dry. Finally, the samples were measured using a Hydroxyproline Assay Kit (Sigma, USA). Hydroxyproline was quantified by 560 nm absorbance with a microplate reader (Molecular Devices, Sunnyvale, CA, USA).

### Measurement of Adenosine-5′-Triphosphate (ATP)

ATP production in the wound beds was measured 1 hr after the first indicated treatments using an ATP assay kit (Promega, Madison, WI, USA), in separate groups of mice. Briefly, the tissues from the wound beds were carefully isolated and homogenized in 1% trichloroacetic acid (sigma, USA). The resultant homogenates were added in duplicate into 96-well luminescence plate containing 100 μl of assay reagent. The luminescence signal was measured with a microplate reader (Molecular Devices, Sunnyvale, CA, USA). Relative ATP levels were normalized to the total protein concentration measured by a Bio-Rad protein assay kit.

### Statistical Analysis

The data were expressed as mean ± standard deviation (SD). Statistical analysis was performed by one-way ANOVA and then Tukey’s Multiple Comparisons tests among multiple groups using Graph Pad Prism 5.0 (Graph Pad Software, La Jolla, CA, USA). Percentage of completely healed wounds was analyzed by two-sample *t*-test (StatPac, Pepin, WI, USA). A p value <0.05 was considered statistically significant.

## Results

### LLLT and CoQ10 additively accelerate wound healing

**Figure 1:**
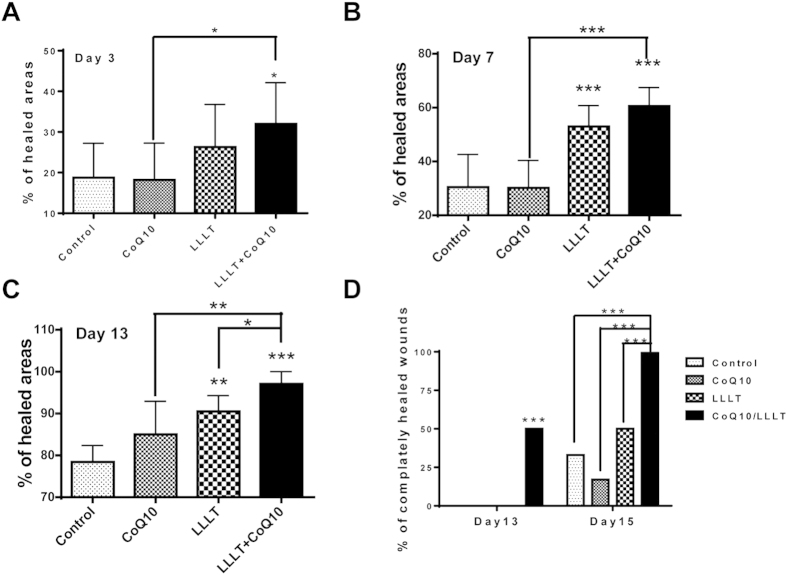
Additive effects of LLLT and CoQ10 on wound healing in diabetic mice. Cutaneous full-thickness wounds were generated in diabetic mice and treated 24 hr later with sham light plus topical vehicle of CoQ10 (control), sham light + topical CoQ10 (CoQ10), LLLT+vehicle (LLLT), or LLLT followed by topical CoQ10 (LLLT+CoQ10). Wound areas were measured with Image J of photos taken at indicated days post-injury (**A–C**). The level of wound healing was defined as percentages of re-epithelialized areas over original wound areas as detailed in Materials and Methods. (**D**) Percentages of wounds with complete healing in each group. Complete wound healing as defined by full epithelialization was tracked in 13 and 15 days. Note: all skin wounds were completely healed in day 15 in LLLT+CoQ10 group only. *p < 0.05, **p < 0.01 and ***p < 0.001 between LLLT+CoQ10 group and all other three groups or between indicated groups. n = 12 in each group for data collected before or on day 7 or n = 6 for data collected after day 7.

**Figure 2:**
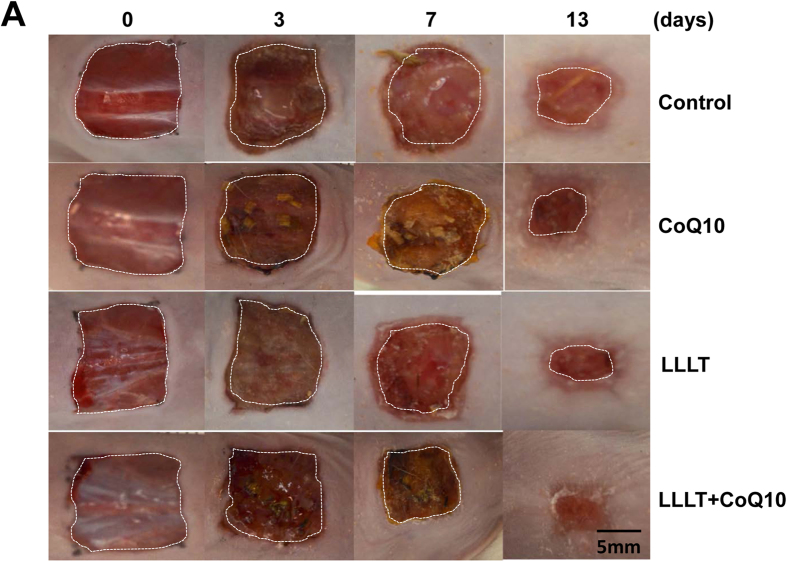
Representative photos of each treatment. Wounds were generated and photos were taken as in Fig. 1. Each row depicts the same wound as it progresses for 13 days. Shown are representative photos from indicated treatments as above. Bar, 5 mm.

To examine wound-healing responses to LLLT, CoQ10, or both, two full-thickness wounds were made in the dorsal skin of each mouse, one for treatment and the other for the corresponding control. In sham-treated controls, the healed area was 19% on day 3, 30% on day 7, and 78% on day 13 (Fig. 1A–C). In comparison, the dual treatment of LLL and topical CoQ10 significantly accelerated healing to 32% on day 3 (p < 0.05), 61% on day 7 (p < 0.001), and 97% on day 13 (p < 0.001). In other words, LLLT followed with topical CoQ10 resulted in 68~103% improvement in wound healing during the first week and more than 24% in the following week. The LLLT plus CoQ10 group outperformed the only LLLT and only CoQ10 groups as well (Fig. 1A–C). While mice treated with LLL healed slower than LLLT+CoQ10 (Fig. 1A–C), they did heal faster than controls in agreement with prior studies showing beneficial effects of LLLT on the wounds of diabetic subjects^2^. For LLLT alone, the healed area was 26% on day 3, 53% on day 7 (p < 0.001), and 91% on day 13 (p < 0.05) as compared to sham-treated controls (Fig. 1A–C). On the other hand, the CoQ10 group did not demonstrate statistical significances in wound healing compared to controls. The difference in the percentages of wound healing was significant between LLLT+CoQ10 and only CoQ10 groups at all experimental days tested, which held the truth between LLLT+CoQ10 and only LLLT group on day 3 and day 13 (p < 0.05), but not on day 7 (Fig. 1A–C). Representative photos following different treatments are shown in Fig. 2, where each row of the figures features the progression of wound healing in a single subject over time, and areas outlined by white dashed lines indicate un-epithelialized areas. Significant reduction in wound area could be seen by the naked eye on day 7 following LLLT plus CoQ10 treatment, as compared to single modality or sham-treated control. Notably, complete re-epithelialization was found in the mice treated with LLL plus CoQ10 by day 13, as shown in the lower right corner of the figure. In accordance with this, about 50% of wounds showed complete healing on day 13 after treatment with LLL followed by topical CoQ10 and all of the wounds were fully healed by day 15 (Fig. 1D). In contrast, none of sham-treated controls or wounds treated with LLLT or CoQ10 alone demonstrated complete wound healing by day 13, and only 2, 1, or 3 out of 6 healed in the controls or wounds treated with CoQ10 alone or LLL alone by day 15, respectively (Fig. 1D). Complete healing of the wounds receiving LLLT in combination with CoQ10 was highly significant on both days 13 and 15 compared with controls or wounds treated with LLLT or CoQ10 alone (p < 0.001), whereas no difference was found in the wounds treated with LLL alone, CoQ10 alone vs. sham treatment.

### Significant increases in hydroxyproline levels by LLLT and CoQ10

**Figure 3:**
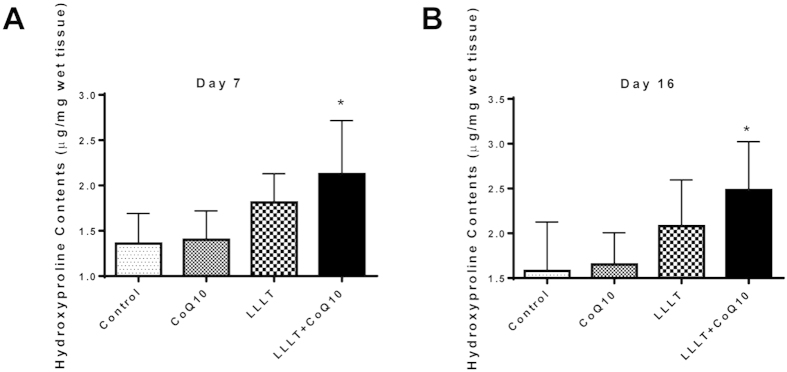
Increased hydroxyproline contents in the wound tissue after treatment with LLLT plus CoQ10. Hydroxyproline contents were measured as μg/mg wet tissue in 7 (**A**) or 16 (**B**) days post-injury. *p < 0.05 between LLLT+CoQ10 group and all other three groups. n = 6 in each group.

Hydroxyproline is a non-proteinogenic amino acid largely restricted to collagen, so its measurement can be used as an indicator of collagen content. The mean hydroxyproline content in the control group was 1.36 ±0.15 μg/mg wet tissue on day 7 and increased to 1.58 ±0.24 μg/mg on day 16. In accordance to additive effects of LLLT and CoQ10 on wound healing, the levels of hydroxyproline were elevated to 2.13 ±0.24 μg/mg from 1.36 ±0.15 μg/mg on day 7 and to 2.48 ±0.22μg/mg from 1.58 ±0.24 μg/mg on day 16 in the LLLT+CoQ10 group, which were more than 50% increases over corresponding control groups (p < 0.05). A high level of hydroxyproline or collagen indicates active healing since collagen increases the strength of the wound and is crucial for many processes at early stages of wound healing. Hydroxyproline values were higher in the LLLT group than in the sham-treated control, but no significant difference could be established between LLLT (1.81±0.14 μg/mg on day 7 and 2.08±0.23 μg/mg on day 16) or CoQ10 (1.40±0.13 μg/mg on day 7 and 1.65±0.14 μg/mg on day 16) and sham-treated controls (Fig. 3).

### Augmentation of cell proliferation by LLLT and CoQ10

**Figure 4:**
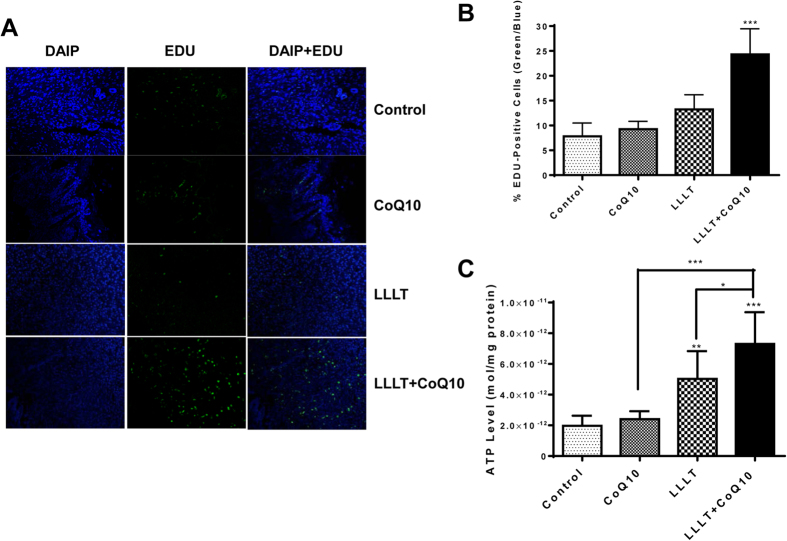
Active cell proliferation in wound tissues following the dual treatment. Cell proliferation was evaluated 7 days after wounds by the EdU assay. (**A**) Confocal microscopy of EdU-stained tissue sections. Green fluorescence represents EdU-positive cells and DAPI stained all cells. Representative results of 36 sections in each group are shown. Magnification, ×20. (**B**) EdU-fluorescence intensity. Percentages of EDU-positive cells over a total number of cells are determined by counting individual cells in 100 microscopic fields selected randomly. ***p < 0.001 between LLLT+CoQ10 group and all other three groups (n = 100). (**C**) ATP levels were measured in duplicate 1 hr after the first indicated treatments. *p < 0.05, **p < 0.01 and ***p < 0.001 between LLLT+CoQ10 group and all other three groups or between indicated groups. n = 8 in each group.

Proliferating cells can be labeled by a thymidine analog EdU and it is simple and robust^21^, allowing consistent results when processing a large number of mouse skin sections. We found that the rate of cell proliferation for controls was 8% on day 7, on average, indicating a rather low level of cell proliferation in the absence of any treatment. LLLT and CoQ10 robustly enhanced cell proliferation rate to 24% (p < 0.001), which represents a 300% increase over controls, while there were no significant differences in the CoQ10 or LLLT alone group compared with controls (Fig. 4 A,B). Thus, comparison between quantities of EdU positive cells in wounds revealed greatly increased cell proliferation following LLLT and CoQ10 treatment compared to single modality treatment or controls (Fig. 4A). Moreover, consistent with additive or synergic improvement of mitochondrial function by LLLT and CoQ10 treatment, ATP production at the wound bed was increased by two times 1 hr after LLLT but it was three times by a combination of LLLT and CoQ10 compared to the control group (Fig. 4C). In contrast, CoQ10 alone didn’t display any significant effect on ATP production (Fig. 4C). Apparently, the level of ATP production at the wound beds was positively and proportionally correlated with the wound healing, similar to previous investigation^22^.

## Discussion

There remain many unmet clinical obstacles to treat diabetic wounds, problems magnified by the consistently rising prevalence of type-2 diabetes. The lack of pharmacologic treatment options creates urgent need for innovative treatment modalities. Poor angiogenesis perturbs oxygen supply in the wound tissues, so targeting ischemia and hypoxia associated with diabetic wounds has led to some success^23^. For example, vascular grafts bypassing arterial occlusions have in many cases promoted healing for ischemic diabetic wounds^24^. Meanwhile, clinicians use hyperbaric oxygen therapy (HBOT) to provide high levels of oxygen to the wound^25^. Despite promising comparative observational studies, HBOT has failed to demonstrate statistical significance in randomized controlled trials^26^,^27^.

Alternatively, early studies have shown that LLLT significantly accelerates wound healing in diabetic rats^28^. LLLT offers a non-invasive and easily performed modality to treat diabetic ulcerations, either alone or as a complement procedure. Its mechanism has been elucidated in part by the ability of LLL to enhance the activity of the electron transport chain and ATP production at mitochondria^9^,^12^,^13^. We recently subjected non-diabetic mice to cerebral injury followed by LLLT, corroborating that LLLT at 830nm enhanced ATP synthesis as well as reduced production of reactive oxygen species (ROS), inflammation, and neuron apoptosis^22^. The beneficial effect of LLLT sufficiently prevented secondary brain injury and significantly diminished tissue loss following traumatic brain injury, particularly in mice with sub-optimal mitochondrial activity^22^. Our investigations further demonstrated that LLL augmented ATP generation, and suppressed ROS formation or apoptosis in the injured brain subjected to hypoxia^29^. The effect of LLLT was significantly bolstered by combining LLL with metabolic substrates like pyruvate or lactate both *in vivo* and *in vitro* as these metabolic substrates can enhance functions of mitochondria even under hypoxic conditions^29^.

Growing evidence points toward a broader connection between mitochondria and diabetes, despite the fact that only an estimated 0.1–9% of diabetic cases involve direct contributions from mitochondrial DNA mutations^30^. Such metabolic dysfunction in diabetics is primarily ascribed to inadequate function of mitochondria that increases ROS production and disturbs cellular signaling involved in wound healing, slowing the healing process^30^,^31^,^32^. Thus we employed LLL and CoQ10, also known as ubiquinone, to substantially improve mitochondrial functions in diabetic wounds. Ubiquinone is both a component in the electron transport chain and, when reduced to ubiquinol, an antioxidant. Oral CoQ10 administration is commonly used to address global mitochondrial dysfunction in diabetics and to improve glycemic control and blood pressure over months of treatment^33^. CoQ10 was also administered intraperitoneally, along with 980 nm LLL treatment to mitigate neuropathic pain in rat daily for two consecutive weeks, but no significant difference was found in the dual treatment relative to single treatment^34^. Likewise, we found no immediate effects on wound healing after administering 1 ml CoQ10 orally every other day for a total of two weeks either alone or in conjunction with LLLT in our initial study (data not shown). Thus we directly applied CoQ10 topically onto wound sites, in hopes that sufficient doses of the quinone could improve mitochondrial function. Indeed, ATP level was elevated additively by LLLT plus CoQ10 as compared to LLLT alone (Fig. 4C), indicating that the dual treatment can better improve mitochondrial function, leading to faster wound healing. Likewise, we previously observed that ATP production at the initial phase of traumatic brain injury was a determinant factor in the control of subsequent wound healing^22^. The level of ATP measured in the early phase of injury was proportionally and inversely correlated with brain tissue loss with a coefficient factor of 0.9^22^. Improved mitochondrial function at the early phase of the wound, manifested by increasing ATP production, may sufficiently prevent cell death while greatly augmenting cell proliferation, leading to accelerated healing. Consistent with this, in our study four groups of mice receiving CoQ10 and LLLT, just CoQ10 or LLLT, or sham treatment controls, whether measured by wound area reduction, collagen production, or cell proliferation, all correlate with ATP production at the early phase of the wound, with the combined CoQ10 and LLLT group demonstrating the highest level of ATP production and fastest wound healing among all treatments tested. Our data clearly demonstrate that LLLT followed by topical CoQ10 has a great potential to be alternative treatment of wounds for diabetic patients either alone or as a complement modality. It merits immediate clinical studies because of its simplicity and super safety.

## Additional Information

**How to cite this article**: Mao, Z. *et al*. Additive enhancement of wound healing in diabetic mice by low level light and topical CoQ10. *Sci. Rep.* **6**, 20084; doi: 10.1038/srep20084 (2016).

## Figures and Tables

**Table 1:** Experiment design.

**Name of the 4 groups**		**Treatments**	**Wounds**	**Animals**	**Sacrificed on day 7**	**Sacrificed on day 16**
Thoracic	Control	sham light+vehicle	12	12	6	6
Lumbar	LLLT	LLLT+vehicle	12			
Thoracic	CoQ10	sham light+CoQ10	12	12	6	6
Lumbar	LLLT+CoQ10	LLLT+CoQ10	12			

## References

[b1] Al-Watban F., Zhang X. & Andres L. Low-level laser therapy enhances wound healing in diabetic rats: a comparison of different lasers. Photomed. Laser Surg. 25, 72–77 (2007). 17508840 10.1089/pho.2006.1094

[b2] Beckmann H., Meyer-Hamme G. & Schröder S. Low level laser therapy for the treatment of diabetic foot ulcers: a critical survey. Evidence-based Complement. Altern. Med. 626127, 10.1155/2014/626127 (2014). PMC3976827 24744814

[b3] Singh N., Armstrong D. & Lipsky A. Preventing foot ulcers in patients with diabetes. JAMA 293, 217–228 (2005). 15644549 10.1001/jama.293.2.217

[b4] Schultz S. . Wound bed preparation: A systematic approach to wound management. Wound Repair Regen. 11, Suppl 1, S1–28. (2003). 12654015 10.1046/j.1524-475x.11.s2.1.x

[b5] Richard J., Sotto A. & Lavigne J. New insights in diabetic foot infection. World J. Diabetes 2, 24–32 (2011). 21537457 10.4239/wjd.v2.i2.24 PMC3083903

[b6] Moulik P., Mtonga R. & Gill G. Amputation and Mortality in New-Onset. Pathophysiology/Complications 26, 491–494 (2003). 10.2337/diacare.26.2.491 12547887

[b7] Driver V. R., Fabbi M., Lavery L. A. & Gibbons G. The costs of diabetic foot: the economic case for the limb salvage team. J. Vasc. Surg. 52, 17S–22S (2010). 20804928 10.1016/j.jvs.2010.06.003

[b8] Eells J. T. . Mitochondrial signal transduction in accelerated wound and retinal healing by near-infrared light therapy. Mitochondrion 4, 559–567 (2004). 16120414 10.1016/j.mito.2004.07.033

[b9] Dungel P. . Low level light therapy by LED of different wavelength induces angiogenesis and improves ischemic wound healing. Lasers Surg. Med. 46, 773–780 (2014). 25363448 10.1002/lsm.22299

[b10] Kim W.-S. & Calderhead R. G. Is light-emitting diode phototherapy (LED-LLLT) really effective? Laser Ther. 20, 205–215 (2011). 24155530 10.5978/islsm.20.205 PMC3799034

[b11] Posten W. . Low-level laser therapy for wound healing: mechanism and efficacy. Dermatol. Surg. 31, 334–340 (2005). 15841638 10.1111/j.1524-4725.2005.31086

[b12] Chaves A. & Piancastelli A. C. C. Effects of low-power light therapy on wound healing : LASER x LED. An. Bras. Dermatol. 89, 616–623 (2014). 25054749 10.1590/abd1806-4841.20142519 PMC4148276

[b13] Silveira P. . Evaluation of mitochondrial respiratory chain activity in muscle healing by low-level laser therapy. J. Photochem. Photobiol. B Biol. 95, 89–92 (2009). 10.1016/j.jphotobiol.2009.01.004 19232497

[b14] Crane F. L. Biochemical functions of coenzyme Q10. J. Am. Coll. Nutr. 20, 591–598. (2001). 11771674 10.1080/07315724.2001.10719063

[b15] Ernster L. & Dallner G. Biochemical, physiological and medical aspects of ubiquinone function. Biochim. Biophys. Acta 1271, 195–204. (1995). 7599208 10.1016/0925-4439(95)00028-3

[b16] Kohli Y. . Effect of hypoxia on acetic acid ulcer of the stomach in rats with or without coenzyme Q10. Jpn. J. Exp. Med. 51, 105–108. (1981). 7277789

[b17] Bentinger M. . The antioxidant role of coenzyme Q. Mitochondrion. 7 Suppl, S41–S50. (2007). 17482888 10.1016/j.mito.2007.02.006

[b18] Choi B. S. . Effect of coenzyme Q10 on cutaneous healing in skin-incised mice. Arch. Pharm. Res. 32, 907–913 (2009). 19557369 10.1007/s12272-009-1613-3

[b19] Watanabe S. . Protective effect of inhalation of hydrogen gas on radiation-induced dermatitis and skin injury in rats. J. Radiat. Res. 55, 1107–1113. (2014). 25034733 10.1093/jrr/rru067 PMC4229932

[b20] Salic A. & Mitchison T. J. A chemical method for fast and sensitive detection of DNA synthesis *in vivo*. Proc. Natl. Acad. Sci. USA 105, 2415–2420. (2008). 18272492 10.1073/pnas.0712168105 PMC2268151

[b21] Bordiuk O. L., Smith K., Morin P. J. & Semenov M. V. Cell proliferation and neurogenesis in adult mouse brain. PLoS. One. 9, e111453. (2014). 25375658 10.1371/journal.pone.0111453 PMC4222938

[b22] Zhang Q., Zhou C., Hamblin M. R. & Wu M. X. Low-level laser therapy effectively prevents secondary brain injury induced by immediate early responsive gene X-1 deficiency. J. Cereb. Blood Flow Metab. 34, 1391–401 (2014). 24849666 10.1038/jcbfm.2014.95 PMC4126101

[b23] Flegg J., Byrne H. M., Flegg M. B. & Sean McElwain D. L. Wound healing angiogenesis: The clinical implications of a simple mathematical model. J. Theor. Biol. 300, 309–316 (2012). 22326476 10.1016/j.jtbi.2012.01.043

[b24] LoGerfo F. W. . Trends in the care of the diabetic foot. Expanded role of arterial reconstruction. Arch. Surg. 127, 617–620 (1992). 1575632 10.1001/archsurg.1992.01420050145019

[b25] Faglia E. . Adjunctive systemic hyperbaric oxygen therapy in treatment of severe prevalently ischemic diabetic foot ulcer. A randomized study. Diabetes Care. 19, 1338–1343 (1996). 8941460 10.2337/diacare.19.12.1338

[b26] O’Reilly D. . Hyperbaric oxygen therapy for diabetic ulcers: systematic review and meta-analysis. Int. J. Technol. Assess. Health Care 29, 269–81 (2013). 23863187 10.1017/S0266462313000263

[b27] O’Reilly D. . A prospective, double-blind, randomized, controlled clinical trial comparing standard wound care with adjunctive hyperbaric oxygen therapy (HBOT) to standard wound care only for the treatment of chronic, non-healing ulcers of the lower limb in patients wi. Trials 12, 69 (2011). 21385365 10.1186/1745-6215-12-69 PMC3061927

[b28] Dancakova L. . Low-level laser therapy with 810 nm wavelength improves skin wound healing in rats with streptozotocin-induced diabetes. Photomed. Laser Surg. 32, 198–204. (2014). 24661084 10.1089/pho.2013.3586 PMC3985531

[b29] Dong Tingting, Zhang Qi, Hamblin, Michael R. & Wu M. X. Low-level light in combination with metabolic modulators for effective therapy of injured brain. J. Cereb. Blood Flow Metab. (2015). Available at: http://www.ncbi.nlm.nih.gov/pubmed/25966949 (Accessed: 13^th^ May 2015) 10.1038/jcbfm.2015.87 PMC4640344 25966949

[b30] Lamson D. W. & Plaza S. M. Mitochondrial factors in the pathogenesis of diabetes: A hypothesis for treatment. Altern. Med. Rev. 7, 94–111 (2002). 11991790

[b31] Senel O., Cetinkale O., Ozbay G., Ahçioğlu F. & Bulan R. Oxygen free radicals impair wound healing in ischemic rat skin. Ann. Plast. Surg. 39, 516–523 (1997). 9374149 10.1097/00000637-199711000-00012

[b32] Panchatcharam M., Miriyala S., Gayathri V. S. & Suguna L. Curcumin improves wound healing by modulating collagen and decreasing reactive oxygen species. Mol. Cell. Biochem. 290, 87–96 (2006). 16770527 10.1007/s11010-006-9170-2

[b33] Hodgson J. M., Watts G. F., Playford D. a, Burke V. & Croft K. D. Coenzyme Q10 improves blood pressure and glycaemic control: a controlled trial in subjects with type 2 diabetes. Eur. J. Clin. Nutr. 56, 1137–1142 (2002). 12428181 10.1038/sj.ejcn.1601464

[b34] Jameie S. B. . Combined therapeutic effects of low power laser (980nm) and CoQ10 on Neuropathic Pain in adult male rat. Med. J. Islam Repub. Iran 28, 58. (2014). 25405124 PMC4219887

